# Serum-Soluble Receptor for Advanced Glycation End Products as a Potential Biomarker in Lung Cancer Patients

**DOI:** 10.3390/jpm16030140

**Published:** 2026-03-02

**Authors:** Emmanouil Panagiotou, Anastasia Georganta, Efstathios Garoflos, Eleftheria Karaviti, Dimitra Karaviti, Athanasios Kontogiannis, Sofia Chorianopoulou, Elias Kotteas, Nikolaos Syrigos, Melpomeni Peppa

**Affiliations:** 1 Oncology Unit, Third Department of Internal Medicine, Sotiria General Hospital for Chest Diseases, National and Kapodistrian University of Athens, 11527 Athens, Greeceeleftheriakrv@gmail.com (E.K.); sophchor@icloud.com (S.C.);; 2 Endocrine Unit, Second Propaedeutic Department of Internal Medicine, Research Institute and Diabetes Center, Attikon University Hospital, School of Medicine, National and Kapodistrian University of Athens, 12462 Athens, Greece

**Keywords:** AGE-RAGE axis, biomarkers, lung cancer, PD-L1 expression, overall survival, soluble receptor for advanced glycation end products, sRAGE

## Abstract

**Background:** Lung cancer (LC) remains the leading cause of cancer-related mortality worldwide. Soluble receptor for advanced glycation end products (sRAGE) has emerged as a candidate biomarker in metabolic, inflammatory, and malignant diseases, although its prognostic significance in LC remains uncertain. **Methods:** Serum sRAGE levels were prospectively measured at baseline and prior to the second cycle of treatment in patients with non-small-cell lung cancer (NSCLC) and small-cell lung cancer (SCLC). Associations of sRAGE with overall survival (OS), progression-free survival (PFS), clinical features, and other biomarkers were analyzed. **Results:** In total, 42 patients were enrolled in this study. sRAGE was detected in 16 patients (38.1%) at baseline and in 15 patients (37.5%) after the first cycle of treatment. Pre-treatment sRAGE levels were strongly correlated with post-treatment levels (Pearson’s r = 0.78; 95% CI, 0.61–0.88; *p* = 4.1 × 10^−9^) and moderately correlated with PD-L1 tumor proportion score in NSCLC patients (Spearman’s ρ = 0.4, *p* = 0.049). Pre-treatment sRAGE levels tended to be higher in patients with indeterminate/high risk of liver fibrosis compared to patients with low risk (Wilcoxon rank-sum test, *p* = 0.041). Post-treatment change in sRAGE levels was correlated with whole blood cell count-derived inflammatory markers. A preliminary association between decreased sRAGE and overall survival in SCLC patients was observed. **Conclusions:** Serum sRAGE shows potential as a blood-based biomarker reflecting metabolic, immune, and inflammatory status in lung cancer, warranting further investigation to clarify its prognostic and therapeutic relevance.

## 1. Introduction

Lung cancer (LC) is the leading cause of cancer mortality worldwide, with approximately 1.8 million deaths every year [1]. It is broadly classified into two categories: non-small-cell lung cancer (NSCLC), which accounts for approximately 85% of all LCs, and small-cell lung cancer (SCLC), a more aggressive and rapidly advancing form [2]. The major risk factors include advanced age, cigarette smoking, environmental pollutants, occupational exposures, and genetic predisposition [3,4]. The introduction of immunotherapy in LC treatment has increased survival in patients treated with immune checkpoint inhibitors (ICIs), although most patients with metastatic disease will, at some point, develop resistance and disease progression [5].

More recently, attention has turned to the roles of advanced glycation end products (AGEs), either endogenously formed or diet-derived, in cellular metabolism, inflammation, and immune response, with potential implications for cancer patients in general, and those receiving checkpoint immunotherapy in particular [6]. AGEs are a heterogeneous group of irreversible adducts generated through non-enzymatic glycation and glycoxidation of proteins, lipids, and nucleic acids during prolonged exposure to reducing sugars (the Maillard reaction) [7]. AGEs are constantly formed in the body, but their formation is greatly accelerated under conditions of increased inflammation, oxidative stress, insulin resistance, hyperglycemia, and dyslipidemia, such as diabetes, obesity, renal failure, aging, neurodegeneration, and cancer, contributing to the pathogenesis of the diseases and their complications [8,9,10,11,12]. In addition to tobacco smoke, diet-derived AGEs, produced during cooking foods as the result of application of heat, are an important exogenous source, contributing significantly to the total AGE burden and exerting their actions in a similar way to those endogenously formed [9,13]. Once formed, intracellular protective systems, tissue macrophages, and other cellular systems endocytose and degrade AGEs via receptor- and non-receptor-mediated pathways, through which they undergo a variable degree of reabsorption, further catabolism in the proximal nephron, and the rest is excreted in the urine [14]. However, in cases of increased AGE formation and/or consumption, these systems are overwhelmed and cannot degrade AGEs in an efficient way.

In particular, the interaction of AGEs with the receptor for advanced glycation end products (RAGE), a multiligand immunoglobulin superfamily receptor abundantly expressed in the lung and on mononuclear phagocytes [15], activates several pathogenic intracellular pathways, including NADPH oxidase-dependent ROS production, NF-κB activation, and MAPK signaling cascades [16], leading to transcription of pro-inflammatory cytokines, adhesion molecules, and survival/proliferation genes. This sustains a self-perpetuating inflammatory loop, as the RAGE promoter itself contains NF-κB-responsive elements, thereby amplifying RAGE expression with continued ligand exposure [17]. The AGE–RAGE axis, therefore, functions as a significant driver of oxidative stress, chronic inflammation, tissue remodeling, and tumor progression [10,18]. To counterbalance these effects, circulating soluble RAGE (sRAGE), composed of cleaved RAGE (cRAGE) and alternatively spliced endogenous secreted RAGE (esRAGE), acts as a decoy receptor, sequestering AGEs and preventing downstream signaling [17,19]. Conversely, high AGE/sRAGE ratios more reliably mark dysregulated AGE–RAGE biology, reflecting increased ligand availability relative to the protective decoy pool [7]. As RAGE and sRAGE are closely linked to inflammation and immune regulation, sRAGE may help identify patients with alterations in inflammatory signaling.

With the expanding use of immunotherapy, biomarkers that reflect the metabolic and inflammatory states of the tumor microenvironment are becoming increasingly important. The leading prognostic biomarker in the field of immunotherapy is Programmed Death Ligand 1 (PD-L1) expression in tumor cells, as detected by immunohistochemistry. However, PD-L1 expression alone presents several problems as a biomarker, as patients without PD-L1 expression still derive benefit from ICIs, while many patients with high PD-L1 expression will eventually develop resistance [20,21]. Furthermore, PD-L1 expression from T lymphocytes located close to the tumor, as well as tumor heterogeneity, may complicate interpretation [21]. Recently, sRAGE was directly associated with upregulation of PD-L1 through the JAK2/STAT3 signaling pathway in models of myocardial ischemia [22]. Therefore, the relationship between sRAGE and PD-L1 expression in NSCLC patients may be important for predicting treatment response with ICIs.

Circulating inflammatory markers, including metabolic and/or circulating whole blood cell count-based parameters, have demonstrated significant promise in observational studies of ICI-treated patients, although they have not yet entered into routine clinical practice [23,24]. Another immunometabolic score, the fibrosis index (FIB-4), which was developed to assess the risk of fibrosis in patients with chronic liver disease [25], has been associated with increased LC risk and may be related to ICI treatment response in this population [26]. Liver inflammation and non-alcoholic fatty liver disease (NAFLD)/non-alcoholic steatohepatitis (NASH) may alter hepatic or systemic immune surveillance in patients receiving ICIs [27]. The AGE/RAGE axis, including serum sRAGE levels, has been associated with the development of NAFLD [28]. Notably, elevated FIB-4 index may be directly associated with circulating sRAGE [29] and PD-L1 expression levels [30], although this has not been previously evaluated in LC patients.

In this study, we evaluated serum sRAGE levels in patients with LC (NSCLC and SCLC) and investigated their associations with patient demographics, tumor characteristics, the presence of circulating inflammatory markers, and survival outcomes.

## 2. Materials and Methods

In this study, we included patients aged over 18 years with histological or cytological confirmation of advanced or metastatic LC that received systemic therapy at the Third Department of Internal Medicine, “Sotiria” General Hospital for Chest Diseases, School of Medicine, National and Kapodistrian University of Athens, Athens, Greece. Patients had to be treatment-naïve in the advanced/metastatic setting, although prior adjuvant or neoadjuvant therapy that had been completed more than 6 months prior to study enrollment was permitted. Information about disease characteristics and patient demographics was collected at study enrollment. Follow-up information about survival outcomes was prospectively collected at regular intervals with in-person or remote patient interviews.

Serum samples were collected from 42 patients at baseline, before treatment initiation, and from 40 patients at the following patient visit, prior to the second treatment cycle. Samples were centrifuged at room temperature at 1100–1300 *g* for 20 min and subsequently stored at −80 °C. Serum sRAGE levels were measured using the Human sRAGE ELISA Kit (FineTest^®^, Cat. No. EH0408, Wuhan, China; sensitivity: <31.25 pg/mL, Intra-Assay coefficient of variation (CV): <6%, Inter-Assay CV: <8%, Recovery range: 93%). Immunohistochemminical PD-L1 expression was determined using the assays VENTANA PD-L1 (SP263) or Agilent PD-L1 IHC 22C3 pharmDx, as part of routine clinical practice, in NSCLC patients. The FIB-4 index was calculated using the formula: FIB-4 = (Age in years × AST level)/[Platelet count × √(ALT level)] (AST, Aspartate Aminotransferase; ALT, Alanine Aminotransferase).

Tumor response was assessed according to the Response Evaluation Criteria in Solid Tumors (RECIST) version 1.1. Progression-free survival (PFS) was measured from the start of first-line treatment until either disease progression or death from any cause. Overall survival (OS) was measured from the start of first-line treatment to death from any cause. The data cutoff date was 14 November 2025.

All statistical analyses were conducted in R (version 4.2.1) with the survival package. Descriptive statistics were used to summarize patient demographics and clinical features. PFS and OS were estimated with Kaplan–Meier methods. Associations between clinical variables and survival outcomes were examined using Cox proportional hazards models. Optimal sRAGE cut-point values were derived by Receiver Operating Characteristic (ROC) curve analysis. The Wilcoxon rank-sum test was used to analyze differences in categorical variables. Pearson’s and Spearman’s correlation coefficients were used to evaluate linear and monotonic associations in continuous variables, respectively. A two-sided alpha level of 0.05 was applied for all statistical tests.

## 3. Results

Of the 42 patients that were included in this study, 32 patients (76.2%) were male and 10 patients (26.8%) were female. Regarding the LC type, 30 patients (71.4%) were diagnosed with NSCLC, while 12 patients (28.6%) were diagnosed with SCLC. Most patients (81.0%) had metastatic disease at the time of treatment initiation. Smoking history was provided by 38 patients; of those, approximately two thirds (65.8%) were current smokers, and the rest (34.2%) were former smokers. All patients enrolled in this study received chemotherapy, with or without ICIs. Seven patients (16.7%) did not receive ICIs: six had stage II–III disease for which ICI therapy was not indicated at the time of study enrollment, and one experienced rapid deterioration in performance status and received salvage chemotherapy. Patient demographics are summarized in Table 1.

Serum sRAGE levels were detectable at baseline in 11 patients with NSCLC (36.7%) and 5 patients with SCLC (41.7%), while post-treatment sRAGE was detectable in 10 patients with NSCLC (35.7%) and 5 patients with SCLC (41.7%). Serum sRAGE measurements for the study population are summarized in Table 2.

**Figure 1:**
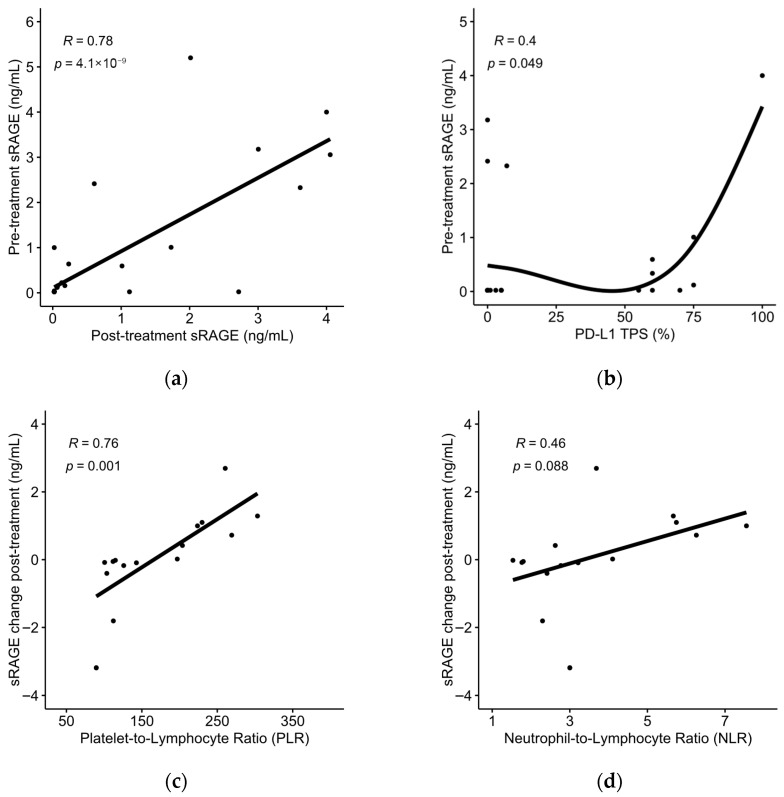
(**a**) Associations between pre-treatment sRAGE and post-treatment sRAGE levels or (**b**) PD-L1 tumor proportion score (TPS); (**c**) associations between absolute change in sRAGE levels and baseline platelet-to-lymphocyte ratio (PLR) or (**d**) baseline neutrophil-to-lymphocyte ratio (NLR).

Pre-treatment sRAGE demonstrated a strong positive correlation with post-treatment sRAGE (Pearson’s r = 0.78; 95% CI, 0.61–0.88; *p* = 4.1 × 10^−9^) (Figure 1a) and a weak positive correlation with PD-L1 tumor proportion score (TPS) in NSCLC patients (Spearman’s ρ = 0.4, *p* = 0.049, n = 25) (Figure 1b). The absolute change in sRAGE levels post-treatment demonstrated a strong positive correlation with the platelet-to-lymphocyte ratio (PLR) at baseline (Pearson’s r = 0.76; 95% CI, 0.40–0.92; *p* = 0.001) (Figure 1c) and a non-significant correlation with the neutrophil-to-lymphocyte ratio (NLR) at baseline (Pearson’s r = 0.46; 95% CI; −0.07–0.78; *p* = 0.088) (Figure 1d).

**Figure 2:**
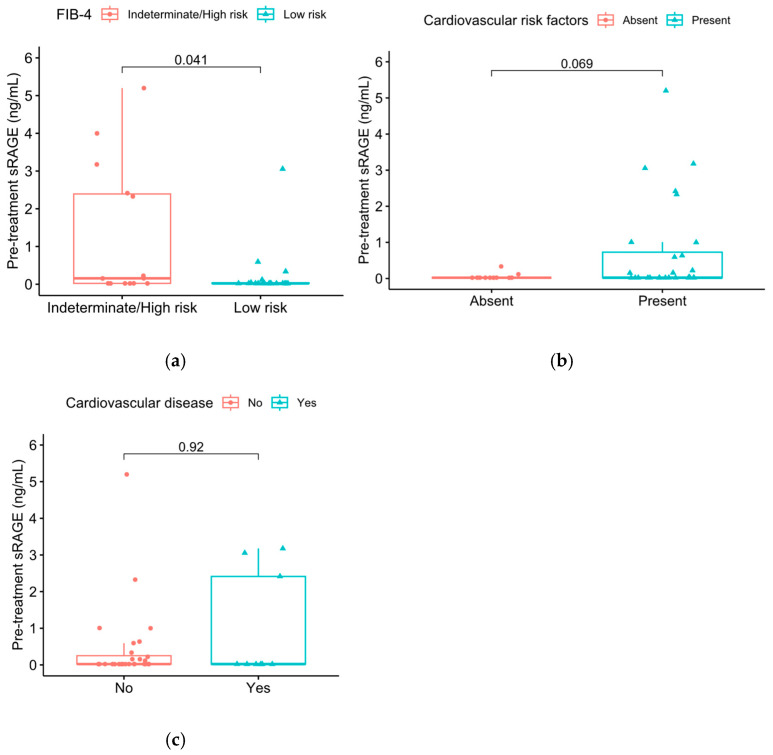
Baseline sRAGE levels in patients with low or indeterminate/high risk of liver fibrosis: (**a**) as defined by FIB-4 index; (**b**) in patients with or without cardiovascular risk factors; (**c**) in patients with or without established cardiovascular disease.

Patients with indeterminate/high risk of liver fibrosis, as defined by FIB-4 index ≥ 1.45, tended to exhibit higher pre-treatment sRAGE levels compared to patients with low risk of fibrosis (Wilcoxon rank-sum test, *p* = 0.041) (Figure 2a). There was a trend towards higher pre-treatment sRAGE levels in patients with cardiovascular risk factors (Wilcoxon rank-sum test, *p* = 0.069), although no significant association was observed between established cardiovascular disease and serum sRAGE levels (*p* = 0.92) (Figure 2b,c). Seven patients (16.7%) received oral corticosteroid therapy at the time of treatment initiation. No significant association was observed between oral corticosteroid therapy and serum sRAGE levels (*p* = 0.8).

Driver alterations were identified in 8 of 19 patients (42.1%) with non-squamous NSCLC, including *KRAS* mutations in five patients, *TP53* mutations in two patients, and an *FGFR2–TACC2* fusion in one patient. No significant association was observed between the presence of driver alterations and serum sRAGE levels (*p* = 0.25).

**Figure 3:**
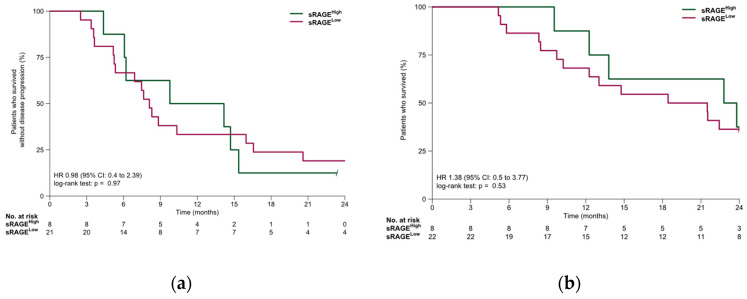
(**a**) Progression-free survival and (**b**) overall survival in the NSCLC cohort.

In the NSCLC cohort, median PFS was 8.3 months (95% CI: 6.9–15.4 months). PFS was similar in the sRAGE-low and sRAGE-high subgroups (8.1 vs. 12.0 months, HR, 0.98; 95% CI, 0.4–2.39; *p* = 0.97). Median OS was 21.5 months (95% CI: 13.0 months—NR). OS was similar in the sRAGE-low and sRAGE-high subgroups (20.0 vs. 23.3 months, HR, 1.38 95% CI 0.5–3.77; *p* = 0.53). Survival outcomes for the NSCLC cohort are summarized in Figure 3.

**Figure 4:**
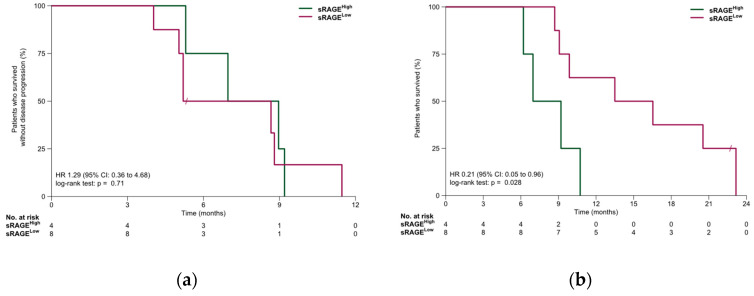
(**a**) Progression-free survival and (**b**) overall survival in the SCLC cohort.

In the SCLC cohort, median PFS was 7.0 months (95% CI: 5.2 months—NR). PFS was similar in the sRAGE-low and sRAGE-high subgroups (6.9 vs. 8.0 months, HR, 1.29; 95% CI, 0.36–4.68; *p* = 0.71). Median OS was 10.3 months (95% CI: 9.1 months—NR). OS was significantly longer in the sRAGE-low subgroup compared to the sRAGE-high subgroup (15.0 vs. 8.1 months, HR, 0.21; 95% CI 0.05–0.96; *p* = 0.028). Survival outcomes for the SCLC cohort are summarized in Figure 4.

## 4. Discussion

In this study, we found that circulating sRAGE is frequently low or undetectable in patients with advanced LC and is variably associated with markers of systemic inflammation, tumor characteristics, and clinical outcomes. Baseline sRAGE levels showed distinct patterns between NSCLC and SCLC, with a prognostic association observed only in the SCLC cohort, while, in NSCLC, sRAGE appeared to be more closely linked to inflammatory indices and PD-L1 expression, rather than survival. Collectively, these findings suggest that sRAGE may reflect underlying immunometabolic and inflammatory processes in LC, with potentially different biological and clinical implications across histological subtypes.

The deregulation of cellular metabolism towards aerobic glycolysis (the Warburg effect) and the development of tumor-promoting inflammation have been widely recognized as hallmarks of cancer [31]. The AGE–RAGE interaction represents a key regulator of glucose metabolism and glycation-associated inflammation [32] and, therefore, constitutes a potential oncogenic driver, which may be important for disease prognosis and therapeutic drug targeting. As RAGE is highly expressed in lung tissue, disruptions in RAGE expression and circulating sRAGE levels are common in different lung diseases [33]. Interestingly, alterations in sRAGE appear to be differentiated in LC compared to other lung diseases. In a study of a Chinese cohort, sRAGE levels were decreased in LC patients compared to tuberculosis patients and healthy controls [34]. Similarly, bronchial sRAGE levels were lower in patients with LC compared to patients with lung infections [35].

A key observation is that, in many patients in our cohort, sRAGE levels were below the assay’s detection limit. Low sRAGE levels are widely reported in conditions of heightened inflammatory stress, including hypertension, coronary artery disease, hyperthyroidism, and rheumatoid arthritis [36,37,38,39], suggesting consumption or impaired generation of decoy receptors when AGE burden is high. Reduced sRAGE has also been reported in various malignancies, such as gastric, colorectal, pancreatic and breast cancer, melanoma, and in patients with bone metastases, where low sRAGE correlates with disease burden, advanced stage, or poor survival [40,41,42,43,44,45]. In addition, genetic alterations in the *RAGE* gene associated with decreased levels of circulating sRAGE have been linked to increased cancer susceptibility [46]; specific *RAGE* polymorphisms may be associated with NSCLC development [47]. Furthermore, NSCLC has been linked to deregulation of cellular metabolism and reduced formation of AGEs, due to changes in oxidative stress exposure, which may reduce circulating sRAGE [48]. Therefore, the fact that many NSCLC patients in our cohort exhibited sRAGE levels below the assay’s detection limit is biologically plausible and consistent with its known suppression in high-inflammatory and high-tumor-burden states. Another possible explanation may relate to differences in the production of sRAGE through proteolytic cleavage and endogenous secretion of RAGE isoforms in LC patients [33,49]; the sensitivity of the ELISA may be different depending on the specific cleavage site or alternative splice variants.

The association between sRAGE and outcomes in LC patients has been less studied compared with its role in other systemic diseases. Previous studies in acute lung injury and acute respiratory distress syndrome (ARDS) suggested sRAGE as a marker of alveolar epithelial injury and poor outcomes [50]. Similarly, in critical illness and sepsis, elevated sRAGE has been linked to disease severity and mortality [51]. To our knowledge, this is the first study to report the potential association of sRAGE with survival outcomes of chemoimmunotherapy in SCLC patients. In the SCLC cohort, increased baseline sRAGE was associated with worse overall survival. While this finding should be interpreted with caution, given the limited number of patients, the potential association with overall survival warrants further evaluation in larger studies of SCLC patients. In contrast, we did not observe an association between serum sRAGE and survival outcomes in NSCLC patients. In one previous study, increased sRAGE at baseline was associated with superior progression-free survival and overall survival in NSCLC patients treated with immunotherapy, with or without chemotherapy [52]. Differences between NSCLC and SCLC may, in part, be attributable to variations in metabolic characteristics in the tumor immune microenvironment [53].

To date, few studies have evaluated sRAGE dynamics or association with prognosis in patients with LC receiving chemoimmunotherapy. In the study performed by Giglio et al., after 3 months of first-line immunotherapy, with or without chemotherapy, sRAGE levels were significantly increased from baseline in patients who responded to treatment compared to non-responders [52]. Interestingly, in another study of patients with lung adenocarcinoma receiving carboplatin and pemetrexed with or without camrelizumab, RAGE mRNA levels decreased after treatment in both cohorts, while patients with radiological response or stable disease had lower post-treatment mRNA RAGE levels than patients with disease progression [54]. While we did not observe any association of early sRAGE dynamics with response rates or survival outcomes in our cohort, post-treatment changes in serum sRAGE were significantly correlated with baseline PLR and trended towards correlation with baseline NLR, suggesting a potential association with inflammatory status, which may merit further investigation. Inflammatory markers derived from peripheral whole blood cell counts have been previously correlated with sRAGE levels in NSCLC patients and in patients with COPD [52,55].

The positive correlation between circulating sRAGE and PD-L1 expression in LC may reflect shared inflammatory mechanisms driven by RAGE ligands, including NF-κB and STAT3 [56], transcription factors integral to PD-L1 expression and regulation [57,58]. In melanocytes and melanoma cells, RAGE has been shown to mediate PD-L1 expression as a response to ultraviolet radiation in a manner dependent on NF-κB [59]. Furthermore, the RAGE ligand High-Mobility Group Box 1 (HMGB1) can activate the PI3K/AKT signaling pathway through interaction with RAGE and, subsequently, promote PD-L1 expression in breast cancer cells [60]. Through its NF-κB-dependent feedback loop, RAGE signaling sustains chronic inflammation, while HMGB1-mediated dendritic-cell activation and chemokine induction help generate an immunologically “hot” microenvironment where PD-L1 is typically elevated [61,62]. Because sRAGE acts as a decoy receptor released in response to increased ligand burden, higher circulating levels may serve as a surrogate marker of intensified RAGE–ligand activity and, consequently, a PD-L1-high inflammatory tumor environment [63]. Thus, sRAGE could indirectly capture RAGE-driven immune activation with potential relevance to predicting immunotherapy responsiveness.

The role of sRAGE in the development and prognosis of cardiovascular disease is debated and may be variable depending on the presence of other aggravating factors. Increased sRAGE has been consistently associated with cardiovascular complications in patients with diabetes mellitus and/or established coronary artery disease [64], but may have a protective role in non-diabetic populations [36,37]. We observed a non-significant trend towards higher serum sRAGE levels in patients with cardiovascular risk factors in our LC cohort. LC-associated inflammation may activate the AGE/RAGE axis in a similar way to diabetes mellitus and other systemic diseases [65]. As a result, elevated sRAGE may be involved in the development of cardiovascular complications in LC patients, which represent a significant cause of mortality in this patient population, including in patients treated with ICIs [66,67]. The potential association between sRAGE and cardiovascular disease in LC patients, including treatment-related cardiotoxicity, warrants further evaluation in larger prospective studies.

We observed increased serum sRAGE levels in patients with intermediate/high risk of liver fibrosis, as assessed by the FIB-4 index, compared to patients with low risk of fibrosis. Similar observations have been made in non-cancer populations, such as participants in the Atherosclerosis Risk in Communities Study [29]. The AGE/RAGE axis, including polymorphisms in the *RAGE* gene, has been implicated in the development of NAFLD [68,69]. The effect of liver inflammation and/or NAFLD on the efficacy of immune checkpoint inhibitors in LC patients is debated, although it may be significant in patients with liver metastases [70,71].

This study has several limitations. Μany sRAGE values were below the assay detection limit, producing zero-inflated distributions and potentially inflating correlation coefficients. Second, the sample size was limited, particularly for subgroup analyses. Third, this study was not powered to evaluate interactions with immunotherapy, which would be of high clinical relevance given the association with tissue PD-L1 expression. Future work should employ assays with greater analytical sensitivity to examine these associations in larger, independent cohorts and explore mechanistic links between the RAGE axis and immune checkpoint regulation.

## 5. Conclusions

In this exploratory study, we investigated serum sRAGE as a potential biomarker in patients with advanced LC. Our findings suggest that baseline sRAGE levels are frequently low or undetectable in LC patients, consistent with high-inflammatory and high-tumor-burden states. In SCLC, elevated baseline sRAGE was associated with worse overall survival, highlighting a possible prognostic role that warrants further validation. In NSCLC, no significant associations with survival outcomes were observed, although sRAGE showed a modest correlation with PD-L1 expression and inflammatory markers, suggesting a potential link to tumor-associated immune activity. Additionally, higher sRAGE levels were observed in patients with intermediate/high liver fibrosis risk and, potentially, those with cardiovascular risk factors, underscoring the broader systemic involvement of the AGE–RAGE axis.

## Figures and Tables

**Table 1:** Patient demographics.

		Overall	NSCLC	SCLC
N (%)		42 (100.0)	30 (71.4)	12 (28.6)
Sex	Female	10 (23.8)	6 (20.0)	4 (33.3)
	Male	32 (76.2)	24 (80.0)	8 (66.7)
Age	≥65	28 (66.7)	20 (66.7)	8 (66.7)
	<65	14 (33.3)	10 (33.3)	4 (33.3)
ECOG PS	0–1	30 (71.4)	21 (70.0)	9 (75.0)
	2+	12 (28.6)	9 (30.0)	3 (25.0)
Smoking status	Current smoker	25 (65.8)	18 (66.7)	7 (63.6)
	Former smoker	13 (34.2)	9 (33.3)	4 (36.4)
Cardiovascular risk factors	Present	28 (71.8)	20 (69.0)	8 (80.0)
	Absent	14 (28.2)	10 (31.0)	4 (20.0)
Disease stage	II–III	8 (19.0)	4 (13.3)	4 (33.3)
	IVA	11 (26.2)	9 (30.0)	2 (16.7)
	IVB	23 (54.8)	17 (56.7)	6 (50.0)
Brain metastases		6 (14.6)	5 (17.2)	1 (8.3)
Liver metastases		12 (28.6)	6 (20.0)	6 (50.0)
Bone metastases		16 (38.1)	13 (43.3)	3 (25.0)
Adrenal metastases		8 (19.0)	5 (16.7)	3 (25.0)
PD-L1 expression (TPS)	≥50%	8 (32.0)	8 (32.0)	N/A
	1–49%	6 (24.0)	6 (24.0)	N/A
	<1%	11 (44.0)	11 (44.0)	N/A
Best Overall Response	CR	1 (2.6)	0 (0.0)	1 (10.0)
	PR	15 (38.5)	12 (41.4)	3 (30.0)
	SD	12 (30.8)	9 (31.0)	3 (30.0)
	PD	11 (28.2)	8 (27.6)	3 (30.0)

Abbreviations: CR, complete response; ECOG PS, Eastern Cooperative Oncology Group Performance Status; NSCLC, non-small-cell lung cancer; PD, progressive disease; PR, partial response; SCLC, small-cell lung cancer; SD, stable disease; TPS, tumor proportion score.

**Table 2:** Serum sRAGE measurements in the study population.

NSCLC Cohort		
	mean [pg/mL] (SD)	
Pre-treatment sRAGE	620.36 (1139.70)	
Post-treatment sRAGE	706.48 (1308.56)	
Absolute change post-treatment	+152.90 (976.53)	
Directional change	n (%)	
Increase	4 (14.3)	
Decrease	6 (21.4)	
No change	18 (64.3)	
	Post-treatment sRAGE	
Pre-treatment sRAGE	Detectable	Undetectable
Detectable	9 (32.1)	1 (3.6)
Undetectable	1 (3.6)	17 (60.7)
**SCLC cohort**		
	mean [pg/mL] (SD)	
Pre-treatment sRAGE	534.30 (1480.33)	
Post-treatment sRAGE	443.27 (912.23)	
Absolute change post-treatment	−182.06 (1865.42)	
Directional change	n (%)	
Increase	5 (42.7)	
Decrease	1 (8.3)	
No change	6 (50.0)	
	Post-treatment sRAGE	
Pre-treatment sRAGE	Detectable	Undetectable
Detectable	4 (33.3)	1 (8.3)
Undetectable	1 (8.3)	6 (50.0)

## Data Availability

The data presented in this study are available on reasonable request from the corresponding author, E.P., due to privacy restrictions.

## Abbreviations

The following abbreviations are used in this manuscript:

LC	Lung cancer
NSCLC	Non-small-cell lung cancer
SCLC	Small-cell lung cancer
ICI	Immune checkpoint inhibitor
AGEs	Advanced glycation end products
RAGE	Receptor for advanced glycation end products
sRAGE	Soluble receptor for advanced glycation end products
cRAGE	Cleaved receptor for advanced glycation end products
esRAGE	Endogenous secreted receptor for advanced glycation end products
PD-L1	Programmed death ligand 1
NAFLD	Non-alcoholic fatty liver disease
NASH	Non-alcoholic steatohepatitis
CV	Coefficient of variation
AST	Aspartate Aminotransferase
ALT	Alanine Aminotransferase
RECIST	Response Evaluation Criteria in Solid Tumors
PFS	Progression-free survival
OS	Overall survival
ROC	Receiver operating characteristic
NLR	Neutrophil-to-lymphocyte ratio
PLR	Platelet-to-lymphocyte ratio
ARDS	Acute respiratory distress syndrome
HMGB1	High-Mobility Group Box 1
